# Ketamine-Induced Neurotoxicity and Changes in Gene Expression in the Developing Rat Brain

**DOI:** 10.2174/157015911795017155

**Published:** 2011-03

**Authors:** Fang Liu, Merle G Paule, Syed Ali, Cheng Wang

**Affiliations:** Division of Neurotoxicology, National Center for Toxicological Research, U.S. Food & Drug Administration, Jefferson, AR 72079, USA

**Keywords:** N-methyl-D-aspartate (NMDA) receptor, ketamine, apoptosis, *in situ* hybridization, microarray analysis.

## Abstract

Ketamine, an N-methyl-D-aspartate (NMDA) receptor antagonist, is widely used for analgesia and anesthesia in obstetric and pediatric practice. Recent reports indicate that ketamine causes neuronal cell death in developing rodents and nonhuman primates. The present study assessed the potential dose- and time-dependent neurotoxic effects and associated changes in gene expression after ketamine administration to postnatal day 7 (PND-7) rat pups.

Pups were exposed to ketamine subcutaneously at doses of 5, 10, or 20 mg/kg, in one, three or six injections respectively. Control animals received the same volume of saline at the same time points. The animals were sacrificed 6 h after the last ketamine or saline administration and brain tissues were collected for RNA isolation and histochemical examination. Six injections of 20 mg/kg ketamine significantly increased neuronal cell death in frontal cortex, while lower doses and fewer injections did not show significant effects. The ketamine induced cell death seemed to be apoptotic in nature. *In situ* hybridization demonstrated that NMDA receptor NR1 subunit expression was dramatically increased in the frontal cortex of ketamine treated rats. Microarray analysis revealed altered expression of apoptotic relevant genes and increased NMDA receptor gene expression in brains from ketamine treated animals. Quantitative RT-PCR confirmed the microarray results. These data suggest that repeated exposures to high doses of ketamine can cause compensatory up-regulation of NMDA receptors and subsequently trigger apoptosis in developing neurons.

## INTRODUCTION

Ketamine is an N-methyl-D-aspartate (NMDA) receptor ion channel blocker that is being widely used in obstetric and pediatric anesthesia. It is short acting and provides rapid dissociative anesthesia followed by rapid recovery. It received FDA approval in 1970 for use as an anesthetic in adults. Initially, interest in the effect of NMDA-receptor antagonists on the developing brain was focused on preventing hypoxic/ ischemic brain damage [1]. Subsequent studies, however, suggested that, in the developing brain, NMDA-receptor antagonists may also have direct neurotoxic effects. After the first report on neurotoxic effects of NMDA-receptor antagonists in rats during the early stage of central nervous system (CNS) development  [2], the possible toxic effects of ketamine on the immature brain have been more extensively explored. It appears that during rapid synaptogenesis, or the brain growth spurt period, neurons are very sensitive to perturbations in their synaptic environment. In rodents, the brain growth spurt period occurs during the first 3 weeks after birth and is characterized by a rapid increase in brain weight and proliferation of astroglial and oligodendroglial cells [3], etc. To address concerns that findings from rodents may not be relevant to humans (the development of the CNS in rodents is quite different from that of humans), nonhuman primates have been used to assess the neurotoxicity ofNMDA receptor antagonists on pre- and postnatal neurons [4, 5]. Results from nonhuman primates that are consistent with rodent findings accentuate concerns about the potential side-effects of NMDA receptor antagonists in humans. Although much evidence now indicates a direct relationship between blockade of NMDA receptors and neurodegeneration, the underlying mechanism(s) remain(s) unclear. It has been postulated that the elevated neuronal cell death induced by ketamine may involve a compensatory upregulation of NMDA receptor subunits and subsequent over-stimulation of the glutamatergic system by endogenous glutamate [4, 6, 7].

DNA microarray technology has been widely used to obtain gene expression profiles and recently has been incorporated into toxicological research. It allows for the simultaneous assessment of alterations in the expression of tens of thousands of genes in a single experiment. Together with other bioinformatics tools, this technology has been widely used to investigate the mechanisms of toxicant action, to search for novel biomarkers of toxicity and to build molecular signatures or models for predictive toxicology [8]. Thus, DNA microarray technology should provide a relatively complete picture of how the NMDA antagonist, ketamine, affects gene expression in developing brains and give clues about the possible pathways through which ketamine induces neurodegeneration. In the present study, we assessed ketamine-induced neuropathological and neurobiological outcomes in infant rats treated with ketamine and used DNA microarray technology to explore associated changes in gene expression.

## MATERIALS AND METHODS

### Ketamine Treatment

Seven-day-old (PND-7) Sprague Dawley (male and female) rat pups were treated with ketamine hydrochloride (Ketaset, Fort Dodge Animal Health, Fort Dodge, IA). Pups were randomly assigned to treated and control groups for histochemical and gene array studies (n = 5/group). All animal procedures were approved by the Institutional Animal Care and Use Committee of the National Center for Toxicological Research and performed in accordance with the Public Health Service Policy on Humane Care and Use of Laboratory Animals. Ketamine was diluted in saline and administered subcutaneously at doses of 5, 10, or 20 mg/kg, in one, three or six injections at 2-h intervals. Control animals received saline at the same time points. Between each injection animals were returned to their dam to help maintain body temperature and reduce stress. All animals were sacrificed 6-h after the last injection by either decapitation for RNA isolation and *in situ* hybridization or *via* transaortic perfusion with 0.9% saline and 4% paraformaldehyde in 0.1 M phosphate buffer (pH 7.2) for morphological studies. 

### Measurement of Ketamine in Rat Brain and Plasma

Rats treated with six injections of 20 mg/kg ketamine or saline were sacrificed at 5 min, 1, 2, 4, 6, and 18 h following the last injection. Blood and brain tissues from frontal cortex were collected for analyses of ketamine levels.

### Electron Microscopy (EM)

The nature of the ketamine-induced neurodegeneration was examined using EM. The frontal cortex was fixed in 4% paraformaldehyde and 0.1% glutaraldehyde in 0.1M phosphate buffer (pH 7.4) and postfixed with 1% osmium tetroxide in 0.1M cacodylate buffer, subsequently washed with 25% and 50% ethanol plus 5% uranyl acetate, followed by dehydration in an ascending ethanol series and embedded in Epon. The frontal cortex thin sections were stained with uranyl acetate and lead citrate and examined at 60 kV using a Philips CM100 electron microscope (FEI Corporation, Eindhoven, Netherlands).

### *In Situ* Hybridization

An oligonucleotide probe complementary to the mRNA encoding the NMDA receptor NR1 subunit was selected based on cloned cDNA sequences. The sequence of the probe used for *in situ* hybridization was as follows: 5’-TTCCTCCTCCTCCTC-ACTGTTCACCTT-GAATCGGCC-AAAGGGACT (this corresponds to a region that is constant across all NR1 splice variants). [^35^S] deoxy-ATP (New England Nuclear, Boston, MA) and terminal deoxy-nucleotidyl transferase (Boehringer Mannheim Corp., Indianapolis, IN) were used to label the 3’ end and attain specific activities of approximately 5–8 x10 cpm/µg. The specificity of the probes has been described by Monyer *et al.*, [9] and Moriyoshi *et al.*, [10]. Coronal sections (10 µm) through the frontal cortex were cut with a cryostat, rinsed in PBS and processed for *in situ* hybridization as described previously [11]. After an overnight hybridization at 41^o^C, slides were washed in 4×, 1×, and 0.1× sodium chloride-sodium citrate solution, dehydrated in 70% ethanol and air-dried. Autoradiography was performed using Kodak (Rochester, NY) NTB3 emulsion; slides were exposed for 3 weeks at 4^o^C. Hematoxylineosin counterstaining was performed to aid in the analysis of the *in situ* hybridization autoradiographs.

### RNA Isolation

Brain tissue from frontal cortex was collected for RNA isolation. Total RNA was extracted using RNeasy^®^ Lipid Tissue Mini Kits (Qiagen Inc., Valencia, CA). The RNA yield was examined spectrophotometrically by measuring the optical density at 260 nm. The purity and quality of the extracted total RNA were evaluated using the RNA 6000 LabChip kit and Agilent 2100 Bioanalyzer (Agilent Technologies, Palo Alto, CA). High quality RNA [with RNA integrity numbers (RINs) greater than 8.5] was used for the microarray experiments and TaqMan gene expression assays.

### Microarray Analysis

To identify signatures in gene expression profiles associated with ketamine treatment, a microarray technique was used to examine the gene expression patterns in PND 7 rat brains. Gene expression profiling was performed with the Illumina Rat Ref-12 Expression BeadChip platform that contains 22,226 probes (Illumina Inc., San Diego, CA). Data from the Illumina were further input into ArrayTrack, a software system developed by NCTR/FDA for the management, analysis, visualization and interpretation of microarray data. Clustering analyses were conducted using ArrayTrack™. Gene function analysis of Gene Ontology was conducted using FatiGO+ (a web-based bioinformatics tool for the functional profiling of genome-scale experiments specifically oriented for the interpretation of microarray experiments) [12] and GOFFA [13].

## RESULTS

### Ketamine Levels in Plasma and Brain

5 min after ketamine injection, ketamine levels in plasma were highest (5.80 ± 3.10 µg/ml) and decreased to 0.01 ± 0.01 µg/ml (mean ± SEM) at 18 h. Ketamine levels in brain tissue were lower than in plasma, but similar to plasma in terms of time course: ketamine levels in brain were highest at 5 min (2.65 ± 1.60 µg/g) after ketamine administration and decreased to 0.03 ± 0.02 µg/g at 18 h. Ketamine concentrations in plasma and brain decreased in a manner consistent with a single elimination process with a half-lives of 0.68 and 0.90 h, respectively [14].

### Ketamine-Induced Neurotoxicity

**Fig. (1):**
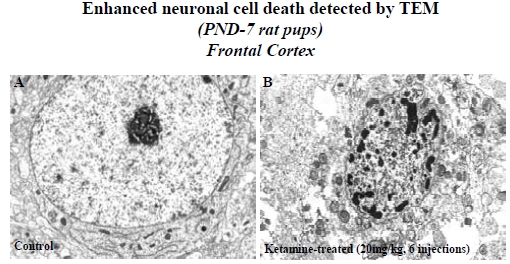
Electron Micrographs (EM) showing a normal neuron in frontal cortex with intact cytoplasm and nuclear membrane from a control rat (PND 7) brain **(A)**. The EM in **(B)** shows the typical nuclear fragmentation representing apoptosis in layers II and III of the frontal cortex from a ketamine- (20 mg/kg × 6 injections) treated rat brain.

At the EM level, DNA degradation and chromosome condensation were observed in layers II and III of the frontal cortex of PND-7 rats after 6 ketamine (20 mg/kg) injections, providing direct evidence of neuronal cell death (Fig. **1B**). DNA degradation and chromosome condensation indicate advanced states of apoptosis. The control animals showed normal cortical neurons with an intact cytoplasm and nuclear membrane (Fig. **1A**). 

No obvious detrimental effects were observed in animals injected with 20 mg/kg ketamine either one or three times, indicating that shorter exposure durations and lower doses were not sufficient to cause neurodengeneration. Likewise, no significant increase in cell death was detected in rat brains exposed to 5 or 10 mg/kg of ketamine in single or multiple injections (three or six times) compared to controls [14]. In the present study, quantitative examinations were carried out on several major brain regions including striatum, hippocampus, thalamus and amygdala. Repeated ketamine injections (20 mg/kg × 6) caused a more than 10-fold increase in neuronal damage in the frontal cortex and around a 3-fold increase in the striatum, hippocampus, thalamus, and amygdala as revealed using immunohistochemical and morphological methods. These data suggest that the frontal cortex is the region most vulnerable to ketamine-induced neurotoxicity during the development [14].

### Ketamine-Induced Alterations in NMDA Receptor Expression (*In Situ* Hybridization Studies)

**Fig. (2):**
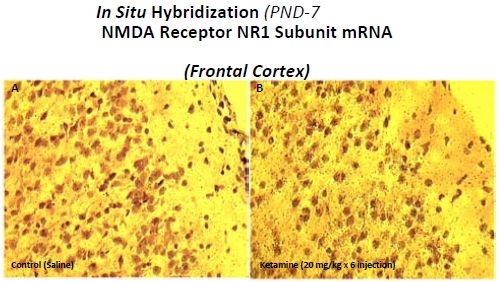
NMDA receptor NR1 subunit mRNA levels assessed by *in situ* hybridization in PND 7 rat brains using a ^35^S-labeled oligonucleotide probe specific for the NMDA receptor NR1 subunit.

NMDA receptor NR1 subunit mRNA was abundant in both control and ketamine-treated (20 mg/kg x 6 injections) rats. The autoradiograph grain density of NR1 mRNA in layers II and III of the frontal cortex was increased in treated rats (Fig. **2**) and the locations of these increases corresponded to the areas where the most damaged neurons were detected. Quantitative analysis of the NR1 *in situ* hybridization signal demonstrated a significant difference between control and ketamine-treated rats (data not shown) and suggests that the neurodegeneration in the frontal cortex of ketamine treated animals is associated with an up-regulation of the NMDA NR1 subunit.

### DNA Microarray Analysis

Microarray data analysis was performed using ArrayTrack™. The criteria for identifying differentially expressed genes (DEGs) were fold-changes greater than 1.4 (up or down) and *P*-values less than 0.05 (in comparison to the control group). There were 819 genes that satisfied these requirements. We performed the functional enrichment of Gene Ontology terms and found that these 819 DEGs were associated with 31 significant terms or pathways including: developmental processes; growth; biological processes; differentiation; behavioral patterns; and receptor activity. In addition, 15 genes relevant to or associated with apoptosis were up-regulated and 17 were down-regulated (Table **1**) [15] and four receptor signaling pathways (glutamate, GABA, dopamine and aryl hydrocarbon) were identified [15].

### TaqMan Gene Expression Assays

The expression levels of genes associated with NMDA receptor subunits were measured with quantitative PCR (Q-PCR) using TaqMan assays (Applied Biosystems). Consistent with the microarray results, the expression of genes associated with NMDA receptor NR1, NR2A and NR2C subunits was significantly higher in ketamine treated animals: no significant effects were observed for genes encoging NR2B or NR2D subunits (Table **2**) [15]. 

## DISCUSSION

To date, data from both rodents and nonhuman primates have demonstrated the neurotoxic effects of ketamine on the developing brain [2, 4, 6, 7]. These findings have raised concerns regarding the safe administration of ketamine in the obstetric and pediatric setting. Further experiments are needed to determine the clinical significance of the animal observations, particularly in light of the fact that the animal studies showing ketamine-induced toxicity have been carried out in intact animals, not in animals undergoing surgical or other traumatic procedures. 

Consistent with the previous data of Ikonomidou *et al.* [2], the present study demonstrated that lower doses and shorter exposure durations of ketamine treatment did not result in neurotoxicity in the developing rat brain. The cell death induced by ketamine was dose and exposure duration dependent. Ketamine levels in plasma and brain were highest soon after the last exposure, decreased rapidly thereafter, and were undetectable within 6 h [14]. An interesting observation is that the time course of plasma and brain ketamine levels do not parallel that for neuronal cell death: when plasma and brain ketamine had decreased to near zero, neuronal cell death was increasing significantly [14]. Meanwhile, increases in NMDA receptor NR1 subunit mRNA levels, as demonstrated using *in situ* hybridization techniques, appeared to be highest in the frontal cortical areas where the most severe neurodegeneration was observed. One explanation for these observations is that the enhanced cell death caused by ketamine is closely associated in time with altered NMDA receptor expression and not *in situ* plasma or brain tissue ketamine levels.

Several lines of evidence indicate that activation of NMDA receptors causes apoptosis and necrosis [4, 5, 16-20]. In nonhuman primates, ketamine-induced neuronal death is characterized by both apoptosis and necrosis [4, 5]. In rodents, however, most evidence indicates that apoptosis is the major cell death pathway caused by NMDA receptor antagonists [17-20]. Several factors may contribute to these differences in ketamine-induced neurodegeneration, such as species, developmental stage at the time of drug exposure, doses and duration of exposure, etc. In the studies described herein, morphological data from electron microscopic (EM) observations showed that neuronal cell death in rats was primarily apoptotic in nature [14]. In addition to the EM data, caspase-3 and Fluoro-Jade C staining support the hypothesis that ketamine-induced neuronal cell death in rats is apoptotics in nature [14, 21].

Based on our morphological observations that the most severe neuronal damage seen after ketamine exposure occurred in the frontal cortex, tissue from that area was selected for RNA isolation and microarray analysis. The microarray data revealed that the expression of 32 apoptosis-relevant genes were altered in ketamine treated rat brains. Apoptosis-related genes have two distinct roles, to either augment or diminish apoptosis and the final fate of a cell is determined by the balance between these two actions. Among the 32 apoptosis-relevant genes, 10 (out of 15) up-regulated genes were pro-apoptotic and almost half of the down-regulated genes were anti-apoptotic [15]. Although microarray data analysis did not detect changes in the expression of some pro-apoptotic genes such as caspase-3, it is likely that changes in gene expression will vary at different time points. Moreover, cleaved caspase-3, which is formed by a post-translational modification of caspase-3, is the active form of casapse-3. Therefore, it is possible that at the transcriptional level, caspase-3 gene expression in ketamine treated rats will be similar to that of control rats.

Some critical changes in gene expression--as determined from the microarray analysis--were confirmed using QPCR. We hypothesized that the cell death caused by ketamine occurred through a compensatory up-regulation of specific NMDA receptor subunits, such as NR1. This up-regulation then makes neurons which possess these receptors more vulnerable to the excitotoxic effects of endogenous glutamate after ketamine has left the system (near zero plasma and brain levels) and, thus, left these receptors uninhibited. The resulting over-excitation of cells harboring upregulated NMDA receptors results in a dysregulation of calcium signaling, increased oxidative stress [19], and activation of the NF-kB signaling pathway [7]. Indeed, *in situ* hybridization, microarray analysis and Q-PCR consistently demonstrated higher NR1 expression in ketamine treated rat brains [15]. In addition to NR1, gene expression for the NMDA receptor subunits NR2A and NR2C was also found to be higher in ketamine treated rats [15]. Moreover, we showed in our previous studies that ketamine-induced neurodegeneration was blocked by NR1 antisense oligonucleotides in cultured rat and monkey neurons [6, 7] providing additional evidence supporting this hypothesis. 

Taken together, the present data suggest that ketamine causes increased apoptosis in developing rat brains in a dose and exposure duration dependent manner. Plasma and brain tissue ketamine levels do not correlate in time with the occurrence of cell death, which occurs after when ketamine levels are quite low. Ketamine administration stimulates the up-regulation of NMDA receptor subunits which may further activate pro-apoptotic genes through various pathways and trigger apoptosis. 

## FUNDING

This work was supported in part by an Interagency Agreement (IAG 224-07-007) between NCTR/FDA and the National Institute for Environmental Health Sciences (NIEHS)/National Toxicology Program (NTP), the Center for Drug Evaluation and Research (CDER)/FDA, and the National Institute of Child Health and Human Development (NICHD).

## DISCLAIMER

This document has been reviewed in accordance with United States Food and Drug Administration (FDA) policy and approved for publication. Approval does not signify that the contents necessarily reflect the position or opinions of the FDA nor does mention of trade names or commercial products constitute endorsement or recommendation for use. The findings and conclusions in this report are those of the author(s) and do not necessarily represent the views of the FDA.

## Figures and Tables

**Table 1.** Apoptosis Related Genes Identified by GOFFA (Ref. [15])

	Gene Symbols	Gene Names
1	*Acvr1c*	activin A receptor, type IC
2	*Ahr*	aryl hydrocarbon receptor
3	*Alms1*	Alstrom syndrome 1
4	*Amigo2*	adhesion molecule with Ig like domain 2
5	*Atp7a*	ATPase, Cu++ transporting, alpha polypeptide
6	*Bnip3*	BCL2/adenovirus E1B 19 kDa-interacting protein 3
7	*Bub1b*	budding uninhibited by benzimidazoles 1 homolog, beta
8	*Cd24*	CD24 antigen
9	*Cdc2a*	cell division cycle 2 homolog A (S. pombe)
10	*Inhba*	inhibin beta-A
11	*Myc*	myelocytomatosis oncogene
12	*Ntf3*	neurotrophin 3
13	*Pak7_predicted*	p21 (CDKN1A)-activated kinase 7 (predicted)
14	*Pdia2_predicted*	protein disulfide isomerase associated 2 (predicted)
15	*Rasa1*	RAS p21 protein activator 1
16	*Tnfrsf11b*	tumor necrosis factor receptor superfamily, member 11b
17	*Unc5c*	unc-5 homolog C (C. elegans)
18	*Agt*	angiotensinogen (serpin peptidase inhibitor, clade A, member 8)
19	*Alb*	Albumin
20	*Apoe*	apolipoprotein E
21	*Bag3*	Bcl2-associated athanogene 3
22	*Cebpb*	CCAAT/enhancer binding protein (C/EBP), beta
23	*Clu*	Clusterin
24	*Cryab*	crystallin, alpha B
25	*Gjb6*	gap junction membrane channel protein beta 6
26	*Hrk*	harakiri, BCL2 interacting protein (contains only BH3 domain)
27	*Igfbp3*	insulin-like growth factor binding protein 3
28	*Inpp5d*	inositol polyphosphate-5-phosphatase D
29	*Jun*	Jun oncogene
30	*Mal*	myelin and lymphocyte protein, T-cell differentiation protein
31	*Rassf5*	Ras association (RalGDS/AF-6) domain family 5
32	*Txnip*	thioredoxin interacting protein

Genes 1-17 were down-regulated and genes 18-32 were up-regulated.

**Table 2.** Selective Validation of the Microarray Results by Q-PCR (Ref. [15])

Gene Symbols	Fold-Change (Q-PCR)	Fold-Change (Microarray)
*Grin1 (NR1)*	1.8*	1.5*
*Grin2a (NR2A)*	1.5*	1.2
*Grin2b (NR2B)*	1.0	0.9
*Grin2c (NR2C)*	1.7*	1.5*
*Grin2d (NR2D)*	1.2	1.1

* P<0.05, as compared to the control.
